# Evaluation of real-time use of electronic patient-reported outcome data by nurses with patients in home dialysis clinics

**DOI:** 10.1186/s12913-017-2377-y

**Published:** 2017-06-26

**Authors:** Kara Schick-Makaroff, Anita E. Molzahn

**Affiliations:** Faculty of Nursing, University of Alberta, Edmonton, Alberta Canada

**Keywords:** Patient reported outcomes (PRO), Chronic kidney disease, Home hemodialysis, Peritoneal dialysis, Electronic patient reported outcomes (ePRO), Patient satisfaction, Quality of life, Symptom assessment

## Abstract

**Background:**

Internationally, the use of patient-reported outcomes (PROs) is increasing. Electronic PROs (ePROs) offer immediate access of such reports to healthcare providers. The objectives of this study were to assess nurses’ perspectives on the usefulness and impact of ePRO administration in home dialysis clinics and assess patient perceptions of satisfaction with nursing care following use of ePROs.

**Methods:**

A concurrent, longitudinal, mixed methods study was conducted over 6 months during home dialysis outpatient clinic visits in two cities. Patients (*n* = 99) provided ePROs using tablet computers when they visited the clinic on two consecutive occasions approximately 3 months apart. Results were scored, printed, and given to nurses before patient appointments. Patients completed satisfaction items from the Comox Valley Nursing Centre Client questionnaire following their appointments. All clinic nurses (*n* = 11) participated and they were each interviewed twice, three months and six months after the start of the study.

**Results:**

The five themes that emerged from the interviews with the nurses include: enhancing focus of the nurses, directing interdisciplinary follow-up, offering support to patients through the process, interpreting results from the visual display, and integrating into workflow.

Scores on the Client Questionnaire suggested that patients believed that they received excellent care (97%), and that the nurses perfectly understood their needs (90.9%). However, their satisfaction with care did not change over time when ePRO data was repeatedly provided to their nurses.

**Conclusions:**

Nurses reported that sharing ePRO data in real-time informed their practice. Although there was no statistically significant change in patient satisfaction scores over time, some patients reported changes and benefits from the use of ePROs. Further research is needed to provide guidance about how ePRO data could enhance person-centered care.

## Background

Internationally, the use of patient-reported outcomes (PROs) such as health-related quality of life (HRQOL) is increasing in clinical settings [1–3]. Patients’ reports of their own health are essential and integral components to providing person-centered care. In people with chronic kidney disease (CKD), who experience many symptoms and impairments in HRQOL, these outcomes are particularly important. These symptoms could be assessed more accurately with use of PROs, and results from PROs could be used for collaborative planning to enhance HRQOL. It is possible that use of ePROs can improve communication between patients and their care providers resulting in better more individualized care and greater satisfaction of patients with their care. Hence, the objectives of this study were to assess patient perceptions of satisfaction with nursing care following use of ePROs and to assess nurses’ perspectives on the usefulness and impact of electronic PRO (ePRO) administration in home dialysis clinics.

Electronic PROs (ePROs) [4–8] offer healthcare providers immediate access to reports [9], and integration of ePROs into routine practice offers instant receipt of results with opportunities to discuss them with patients [10, 11]. Researchers have found that this *enhances communication and effectiveness* of the multidisciplinary team [6, 9, 12]. With a few exceptions, the usefulness and impact of ePROs for patients themselves, or for nurses, has rarely been explored.

There has been conflicting evidence relating to the benefits of use of PROs in clinical practice. For instance, in a qualitative study, Wolpert et al. [13] sought the views of young people, mothers and clinicians about use of PROs in mental health and diabetes services. While clinicians perceived that PROs offered opportunities for *more individualised care*, patients were concerned that treatment would be prioritized over therapeutic relationships. Patients were also concerned about how their results would be used, and questioned the safety of revealing their satisfaction with care in case it reflected poorly on their care providers. In a systematic review of the impact of PRO use on patient outcomes, Boyce and Browne [14] found weak evidence to support the claim that providing PRO feedback to healthcare professionals positively impacted patient outcomes. These two studies raise the question: how beneficial are PROs to patients?

The potential benefits of the use of PROs may be influenced by how healthcare providers use PROs. In a theory-driven approach, Greenhalgh [15] described a taxonomy of applications for PROs in clinical practice that included their use as: tools for screening or monitoring, methods to promote person-centered care, decision aids, strategies to facilitate communication in multidisciplinary teams, and means of monitoring quality of care. In cancer care, Greenhalgh et al. [16] qualitatively studied how physicians referred to PROs in their patient consultations. They found that while use of PRO data may have enabled patients to elaborate on their concerns, physicians did not always know how to respond. Like Edbrooke-Childs et al., [17] Greenhalgh et al. used their research to inform the development of training materials for doctors about when and how to use these instruments. Takeuchi et al. [18] also recommended training for physicians in their follow-up on use of PROs. In their study on patient-physician communication, they found that clinicians tended to focus on symptom intervention, but not on patients’ reports of functional concerns. While nurses’ use of PROs has not been studied in detail, Hilarius et al. [10] found that incorporating HRQOL assessments in daily nursing practice increased communication related to HRQOL issues and significantly improved nurses’ awareness of patients’ experiences of pain, level of functioning, and overall quality of life (QOL).

While there are numerous studies involving use of PROs in CKD and transplantation [19–21], there is a significant gap in our understanding of *how nephrology practitioners use PROs in patient care* [22]. This gap exists despite the fact that: a) nephrology experts agree that person-centered care is a priority both for quality improvement and research [23–26]; and b) QOL has been identified as the health outcome that is most valued by CKD patients, and is most useful in dialysis care assessment [25, 27]. To our knowledge, kidney patients’ views on the usefulness of PROs in their care have not been examined. Thus, the focus of our paper is not on PRO results, but rather on nurses’ and patients’ assessment of real-time use of ePROs. The objectives of this concurrent, longitudinal, mixed-methods study [28, 29] were to assess nurses’ perspectives on the usefulness and impact of ePRO administration in home dialysis clinics and assess patient satisfaction with nursing care following use of ePROs in the clinics.

## Methods

### Study design

We employed a concurrent, longitudinal, mixed-methods research design [28, 29] with qualitative interviews of nurses and completion of a satisfaction survey by patients.

### Participants

The quantitative ePRO data was gathered from patients who attended outpatient home dialysis clinics in two cities on the west coast of Canada over a 6-month timeframe. Letters were sent to all patients who participated in the pilot phase of the project [30], inviting them to again participate and encouraging them to arrive early. Inclusion criteria were: being on dialysis at home (either peritoneal or hemodialysis), over 19 years old, and willing to participate. Exclusion criteria included lack of proficiency in English, inability to read, moderate or severe cognitive impairment, or medical crisis.

All nurses in both clinics were invited to participate in the study. They learned about the project through presentations to clinic staff. None of the nurses knew members of the research team before the interviews and learned about the purpose of the study through the presentation.

### Measures and measurement

Three survey instruments were used to collect data for the larger study: 1) The Edmonton Symptom Assessment System for renal patients (ESAS-r:Renal) [31–33]. 2) The Kidney Disease Quality of Life-36 (KDQOL-36) [34]. (ESAS-r:Renal and KDQOL-36 data are not reported in this article) 3) And 14 satisfaction items that were selected and adapted from the Comox Valley Nursing Centre Client Questionnaire [35], previously tested for construct validity and internal consistency [35]. This measure was selected to assess whether patients were satisfied with aspects of their nursing care, including the use of ePROs [29].

Two ePROs were designed as apps to be run using the FileMaker Go iPad app using iOS 7. The KDQOL-36 was completed online using *KDQOL Complete* (provided by Medical Education Institute [36]). Further details about the technology, design, measurement equivalence [37], and internet access are outlined in an article on the pilot phase of the study [30].

### E-PRO intervention and procedure

Patients typically attend a clinic every 3 months; all home dialysis patients from these two clinics were invited to participate by providing ePROs on two consecutive occasions. A $10 gift card from a grocery store was provided both times they participated.

In order to keep the clinics on schedule, participants were asked to arrive 10–15 min early to participate in the study so as to not delay appointments. When patients came for their regularly scheduled appointments, those who met the inclusion criteria were invited to participate by a third party. If the patient was interested, a research assistant in the waiting room obtained informed consent and showed them how to use the tablet computer (iPad™). No other assistance was provided in completing the measures.

**Fig. 1:**
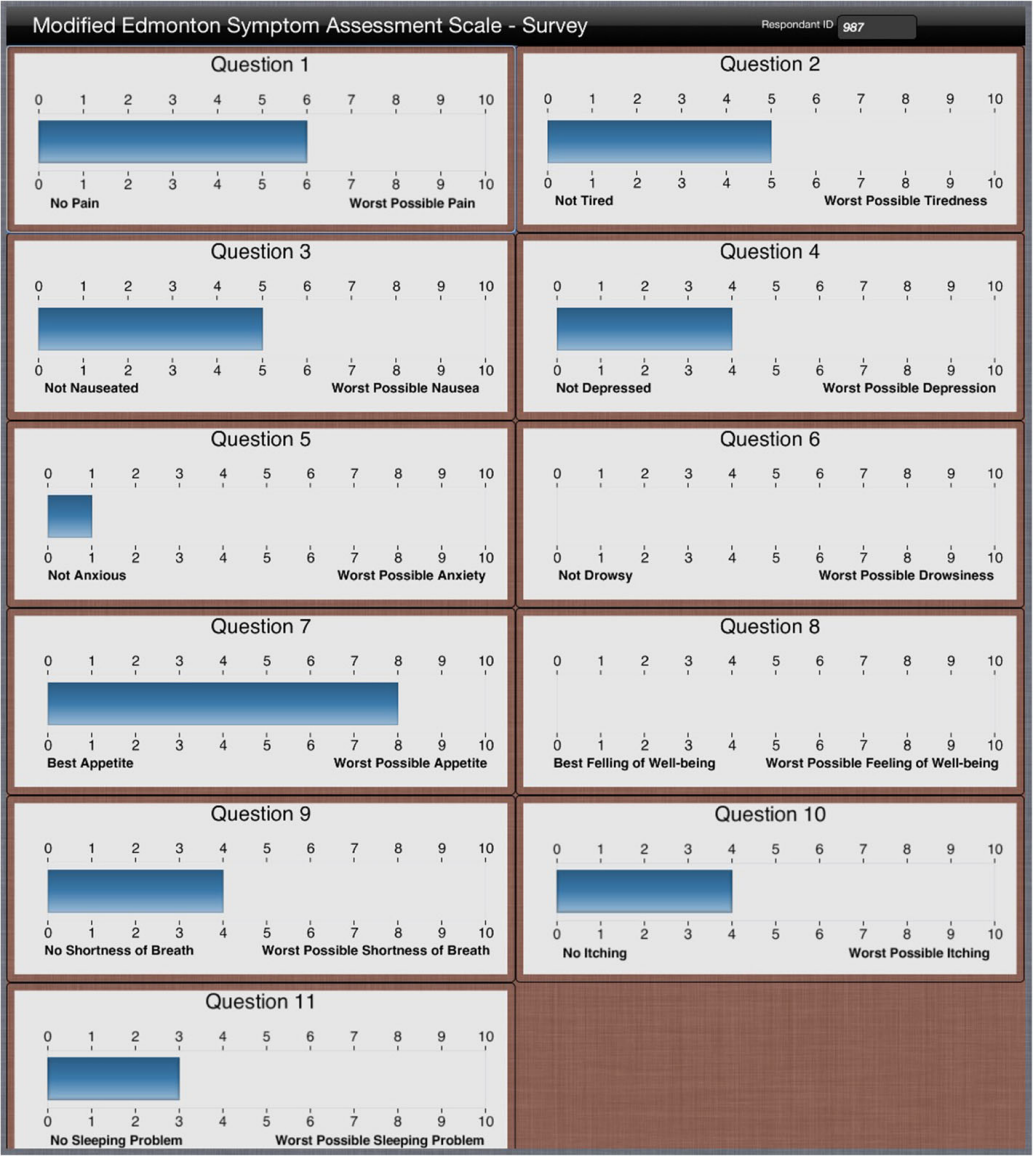
Example of Edmonton Symptom Assessment System renal (ESAS-r:Renal) App Results Display

After completion of the KDQOL-36 and ESAS-r:Renal on the tablet computer, results were printed for the nurses who reviewed the graphically depicted scores with the patients (see Fig. 1 for sample display). Nurses were instructed to review the ePRO scores with the patients and discussions focused on the interpretation of results. They had received education about use of the ePROs, but specific norms or guidelines for responses to scores were not suggested. Patients were provided with their own PRO results, as well as general patient education materials available from the KDQOL-Complete platform if requested (see https://www.kdqol-complete.org/pdfs/KDQOL-Complete-Brochure.pdf).

**Fig. 2:**
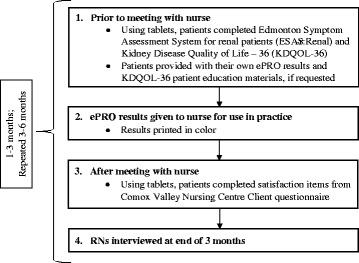
Project Timeline

Following their appointments, patients completed the Client Questionnaire on the tablet computer. This measure was selected to assess whether patients were satisfied with aspects of their nursing care, including the use of ePROs [30]. (See Fig. 2 for summary of procedures used).

### Data collection from nurses: Qualitative component

Interviews were conducted by two female research assistants, both master’s prepared nurses with prior qualitative research experience who were enrolled in doctoral programs. All nurses in the two clinics were invited to participate in two interviews, one mid-way through the study (3 months) and another at the end of the study (6 months). All 11 clinic nurses participated. One nurse was not available for the 2nd interview; in total, 21 interviews were conducted. Interviews ranged in length from 30 to 60 min, and they were conducted in a private location in a clinic at a time convenient for the nurses, often on a lunch break or after work. The interviews were semi-structured (not pilot tested) and started with a guiding question such as, “Can you please describe your experience with use of the ePROs in the clinic?” Nurses were asked to provide examples from their practice of using the ESAS-r:Renal and/or KDQOL-36. In the second interview, nurses were asked to describe examples of how they may have initiated interdisciplinary follow-up based on a patient’s ePRO responses, how their practice may have been affected by receiving ePRO results, what the perceived benefits were for patients, and how they would like ePRO information to be integrated into existing and future work structures. Participants received a $10 gift card for a grocery store as a token of appreciation. Interviews were digitally recorded and transcribed verbatim by a professional transcriptionist. The interviewer took field notes regarding important issues for follow-up in the second interviews or in analysis.

### Qualitative component: Data analysis and rigor

Qualitative data analysis was undertaken using interpretive description, a qualitative methodology used in applied clinical fields [38, 39]. An inductive approach to analysis was employed and data were saturated. The first author, assisted by two research assistants, conducted the analyses. To facilitate the analysis, quotes were highlighted in the electronic transcripts, reflexive notes were created, and quotations were organized into groupings. An audit trail was maintained tracking the analytic decisions. Preliminary findings were shared with nurse participants, and they were invited to provide either written or verbal feedback.

### Quantitative data analysis

Descriptive statistics (means, standard deviations) were calculated for each satisfaction item. Paired t-tests and Hotelling’s T-square (MANOVA) were used to compare changes in each satisfaction item over time. With Hotelling’s T-square, the item “satisfaction with help received” was held as the fixed variable. SPSS (version 21) was used.

### Ethical considerations

IRB approval was obtained from the University of Alberta Health Research Ethics Board (Approval # Pro 00040538) and the Vancouver Island Health Authority (Approval # H2013–065) for all components of the study. All participants provided a written signed informed consent, verbally confirmed at the start of the study and prior to each interview or survey. Consent for publication of individual data was obtained from all participants.

## Results

Characteristics of the patient participants are presented in Table 1. The mean age was 67 years (range from 34 to 88 years), 83% identified as Caucasian, 68% were retired, and 86% were on peritoneal dialysis. Forty-two percent reported previous tablet use.

**Table 1:** Characteristics of patient participants

Clinical characteristics	
Gender (M/F)	66 (67%) /33 (33%)
Age, years	67 ± 12
Diabetes	28 (28%)
Urban / rural	57 (58%) / 42 (42%)
Ethnicity	
Caucasian	82 (83%)
Asian	4 (4%)
African American	1 (1%)
First Peoples	4 (4%)
Pacific Islander	1 (1%)
No data	7 (7%)
Employment Status	
Retired due to age	45 (45%)
Retired due to disability	23 (23%)
Medical leave of absence	8 (8%)
Unemployed	2 (2%)
Employed full time	6 (6%)
Employed part time	6 (6%)
Homemaker	1 (1%)
No data	8 (8%)
Dialysis modality	
Peritoneal dialysis	85 (86%)
Conventional home hemodialysis	9 (9%)
Daily home hemodialysis	4 (4%)
Nocturnal home hemodialysis	1 (1%)
Previous use of tablet computer	
Yes / No	42 (42%) / 57 (57%)

Values are n (%) or mean ± SD

On average, the 11 clinic nurses had 19 years of nursing experience, 14 years of experience working in renal settings (other than home dialysis), 4 years of experience in home dialysis, and national certification in nephrology nursing (55%). Most held a RN diploma (64%) and 46% worked permanent part-time. In Table 2, additional characteristics of the nurse participants are provided.

**Table 2:** Characteristics of RN participants

Demographic characteristics	
Gender (F/M)	11 (100%) / 0 (0%)
Average age ± SD	46 ± 8
Education	
RN diploma	7 (64%)
Bachelor of Nursing	4 (36%)
Canadian Nurses Association certification in nephrology nursing (CNeph)	6 (55%)
Position	
Permanent full-time	3 (27%)
Permanent part-time	5 (46%)
Casual	3 (27%)
Years of nursing experience	
1–10	3 (27%)
11–20	3 (27%)
21–30	5 (46%)
Years of nursing experience in renal settings (other than home dialysis)	
1–10	3 (27%)
11–20	5 (46%)
21–30	3 (27%)
Years of nursing experience in home dialysis	
1–5	7 (64%)
6–10	4 (36%)

Values are n (%) or mean

### Qualitative component: Nurse interviews

Themes identified in the nurse interviews include: provides focus: “be patient-centred”; directs interdisciplinary team follow-up: “it’s a team thing”; offers support: “brings an awareness”; visual display: “it jumps out at you”; and integration with workflow: “long-term picture”. The themes are described below and additional sample quotes are provided in Table 3.

**Table 3:** Sample Quotes re. Usefulness and Impact of ePRO Collection for Nurses

ePRO data was used to:	
Provide focus: “Be patient-centred”	**“**I look to see what is a problem. I ask the patient, repeat the question to the patient, and get them to give me their answer of why they put what they put - just to make sure that they didn’t misunderstand it, misconstrue it, or do whatever, and maybe there’s things in there that um they don’t like to discuss, but at least we can see first-hand what the problems are.” (Nurse 1) “It’s not just symptoms, it’s what’s going on in the patient’s life and addressing them. They are not just a symptom checklist; they’re (pause) - they’re a person that goes through stuff in life and with whatever they’re going through in life they are also living with kidney failure.” (Nurse 3) “Because it [ePROs] would kind of guide the direction of the clinical assessment. And then when we go through the rest of the clinical assessment, all of this is on there and then you can just breeze through it quickly. But if there was something that was triggered here then you kind of go into it a little bit more.” (Nurse 2) “Um well, I think it [ePROs] definitely points out something that is hidden, so it is definitely showing there’s things that we are completely missing - like we’re totally missing ‘x’ with this person and it’s really important to them even though we’re trying to ask them those questions but we’re not getting that information. That’s been really good about this.” (Nurse 5)
Direct interdisciplinary team follow-up: “It’s a team thing”	**“**I think I bring things up verbally as well to the nephrologist if I had some, you know, some area of concern. I would talk to the nephrologist prior to the patient going in to see them because you may not get all your notes written down.” (Nurse 4) “Ah, well, I guess it depends on like if it’s a depression issue, ahm we talk to [the social worker] about it. If it’s maybe an appetite or nausea issue, it might be a dietician thing that they could help with. So we communicate with our team quite a bit - especially [the dietician].” (Nurse 2) “The appetite and nausea, I touch on it but I almost always, you know, say ‘Well of course we’ll speak to the dietician.’ She’s the expert. And I encourage people with psycho-social issues, I encourage them to speak to the social worker, but I don’t cut them short, I don’t. You know, I give them some airtime because they’ve privileged me with the information, I’m not going to minimalize it in any way. I will hear their concern a bit and touch on it, but yes, of course I’ll encourage them to talk to [other clinicians].” (Nurse 6)
Offer support to patients: “Brings an awareness”	“I think it’s improved probably the interactions that we have in our clinic when these patients are, you know, filling out their surveys and are getting these visual tools … you can’t help but look them over and over and address things.” (Nurse 8) “Well I think it’s always beneficial for the patients to be aware of their health and what they can do and make changes to improve it. I mean having them involved and seeing them, writing down a score, I think it’s another (pause) visual so they can say, I feel that - I think that helps them understand. I mean it’s like getting your lab work, you know, you see the numbers.” (Nurse 4) “When they’re sitting in the waiting room, filling in [the ePROs], they can be a bit more honest with themselves and then that is very revealing to the healthcare providers.” (Nurse 6) “So it’s just, to me it’s a tool to try to help focus on trouble areas but knowing a person may or may not be comfortable discussing things with the nurse…I’ve heard people say to me that they don’t want to disappoint me. I’ve been their trainer, their educator, and my role with them has been that way even though I feel like I have a good rapport with the person and spent lots of time with them, they may not have told me anything about their feelings of anxiety and depression.” (Nurse 5)
Used of ePRO data was impacted by:	
Visual display: “It jumps out at you”	**“**I think I do like having a big box of colours cause it draws your eyes to it quicker than maybe, you know, the circling the symptom checklist [on paper]. I think just in the drawing, you know, you focus to the colours, big bars, they kind of stand out more.” (Nurse 4) “It gives nurses a visual tool - that’s what it is.” (Nurse 3) “The one thing that I found was the questions, like these were very small…..Just because the thing that you see is “question 7” but “question 7” doesn’t mean anything to me cause I don’t know it. The symptom is under that typing, until I look. So that was just the one thing I found a little bit small and sometimes sort of like, what am I looking at?" (Nurse 7)
Integration with workflow: “Long-term picture”	“I’d like to see trends, right, like I would like to see maybe not only this [ePRO results], but a printout of, you know, what are the trends in their pain, on an upward or downward? Yeah, I think that’s important, right.” (Nurse 9) “If we’re not getting a report to say where the trends are, but they are at their end of life, you know. So we’re supporting them. And at what point do they say, ‘I’ve had enough?” You know, but we are aware of them…When do we say, or they, say it’s enough?” (Nurse 4) We’re “using the [ePRO] data to supplement the charting.” (Nurse 8) “Cause it makes no sense to do this if it is a tool that helps us at the moment, but I want to see that moment over time just like any lab record, I want to see it over time, over the year.” (Nurse 4)

#### Provides focus: “be patient-centred”

Many nurses said that they looked at the ePRO data immediately so that they could focus on the priorities or concerns of the patient. Nurse 5 said, “I look at it [ePROs] before I start the interview. I focus on it first - those are the things that are important to the patient, rather than focus on what's important to me.” Keeping the patient “in the middle of the visit” was a way for nurses to “be patient-centred.” The nurses emphasized the need to address patients “as a whole person” while simultaneously focusing on a specific physical symptom or a psychosocial issue.

The nurses saw the ePROs as “tools” that helped them focus their nursing assessment. As Nurse 2 said, the ePRO results “trigger us, as nurses, to dig into it a bit more, ask more questions, and maybe investigate a little bit more.” As the nurses narrowed their clinical assessment, the ePRO data helped them identify areas that otherwise might have been missed. Nurse 5 said “I might not ever ask them about something that was rated really high on the survey…if I hadn't seen that, I probably would not have focused in on that one item.” They believed that by focusing their assessments, they saved time and altered how they did their work. And as Nurse 8 commented, “has it changed your practice? I think it does.”

#### Directs interdisciplinary team follow-up: “It’s a team thing”

The nurse participants worked in interdisciplinary home dialysis clinics that involved social workers, dietitians, and nephrologists. Each patient came to an outpatient clinic for 2–2.5 hours and met individually with each clinician. Patient-nurse interviews averaged 20 minutes, but if required, they ran up to 1 hour. While the nurses emphasized they had always worked collaboratively with their colleagues, they spoke in great detail about how they highlighted patients’ priorities to their colleagues for interdisciplinary follow-up. They communicated significant findings from the ePRO data with their team members either verbally, in writing, or via email.

Nurses included patients in this team follow-up. As Nurse 4 said, “I'll give a verbal report with the patient too because they're part of that discussion and things that we have highlighted.” The clinic nurses also encouraged patients to initiate conversations about their own needs with any one of the other clinicians. Nurse 6 said, “people will speak to who they're comfortable speaking to.” Seeing patients as an integral part of the team, Nurse 1 explained, “because it's a team thing we need - everybody has to be on the same page so we can all help the patient work through the particular concerns that they're having.”

#### Offers support: “brings an awareness”

The nurses believed that the process of completing the ePROs provided support to patients. In the clinic waiting rooms, completion of the ePROs sparked conversations between patients, and between patients and family members. Nurse 8 recounted, “Sometimes you see the dynamic in the waiting room where the spouse will be making comments, looking over their shoulder saying, ‘No, I wouldn't put that!’” The nurses also believed that this process facilitated patient engagement in their own personal health. Nurse 10 said, “I think it brings an awareness to them in that moment and makes them think about each individual thing so it's like right at the front of their brain when they go in to talk to the nurse.” The nurses perceived that this “patient-driven” process provided patients with “support”, “acknowledgement”, “improved quality of life”, and the sense of “feeling heard.”

The nurses believed that it may have been easier for the patients to initially complete the ePROs rather than talk about the items, especially for challenging topics such as depression, anxiety, sex, or pain. Nurse 2 wondered, “maybe they're embarrassed a little bit or it's easier to put it on a screen maybe than talk about it?” The nurses thought that the numeric scores offered “safety” to the patient to express concerns in numbers and not words. Nurse 3 explained, “It's easier for them (pause) on a scale like than to say, ‘like this is really bothering me’ because sometimes when they use descriptive words it doesn't sound as (pause) bothersome as when you put in a number.” The nurses believed that many patients downplayed their concerns verbally, often saying “I’m fine, I’m fine”, and then scoring these issues much higher on ePROs.

#### Visual display: “it jumps out at you”

The nurses emphasized that their use of ePRO data was impacted by how the data were visually displayed. ePRO results were printed in color: blue bars for the ESAS-r:Renal (see Figure 1), and red, yellow, and green pie charts for the KDQOL-36 domains. Nurse 11 explained, the colors “gave me a quick glance of things I should address.” And Nurse 6 said, “it jumps out at you more 'cause of the highlighting.”

Many nurses offered suggestions for how the ePRO result display could have been presented in a more user-friendly format. Some requested that data be displayed in one column (instead of two), and others wished that the font had been larger. The visual display of the results was very important to their ability to interpret results quickly and integrate them into their clinical assessments and follow-up.

#### Integration with workflow: “long-term picture”

The nurse clinicians explained that their use of the ePRO data was impacted by whether or not the data was integrated with their workflow. They wished that ePRO data could be displayed longitudinally, in graphs, to see trends over time. Instead, they found themselves reading through charts to gain historical perspective. “You kind of like to have a general long-term picture, not just like one, 20-minute interview” (Nurse 3). Seeing those trends were particularly important to nurses caring for patients whose health was declining or were “at their end of life.”

The nurses admitted that they spent more time focusing on the ESAS-r:Renal (rather than the KDQOL-36) because they were familiar with it and it was required to be part of the health authority data registry. The nurses’ lack of familiarity with the KDQOL-36 necessitated that they spend more time with it, and sometimes that time wasn’t available. Nurse 8 explained, “If I had two things to look at, I'm going to choose the one that I'm familiar with because it takes me more time to figure that [KDQOL-36] out.” The nurses also pointed out that there was a lot of “overlapping” between the mandated ESAS-r:Renal, their nursing assessment checklists, and unit charting documents. Nurse participants were hopeful that when ePRO results were shared between clinicians, then they could “reduce the number of questions” that were repeatedly asked to each patients during each clinic visit.

### Quantitative component: Satisfaction with care

Over the 6-month period, 126 home dialysis patients attended the two outpatient clinics. Ninety-nine patients participated (79% response rate) during the first 3 months. Sixty-nine patients participated at the second point of data collection, 3 months later. Reasons for not participating a second time included not having a second scheduled visit (*n* = 18), declining health or death (*n* = 9), moving to a different city (*n* = 1), changing appointments to telehealth modality (*n* = 1), and withdrawing from the study (*n* = 1).

At the second point of data collection (3 months later), after providing ePROs, patients reported being very satisfied with the amount of help they received (86.4%), as well as the services they received at the clinic (92.4%). They believed that the services they received helped them deal more effectively with their concerns or problems (71.2%). Patients rated the quality of the services they received as excellent (97%), and reported that the nurses perfectly understood the kind of help that they wanted (90.9%).

Patient participants were asked to report on the impact of providing ePROs to their nurses. The second point of satisfaction data collection, 3 months later, is presented in Table 4. Forty-one and a half percent (41.5%) of patients identified that providing ePROs to their nurses was “just the same” as in previous clinics, and 44% reported that their experiences of care were also “just the same”. Also, some patients reported that they felt a great deal healthier (31.8%), more hopeful (31.8%), and more supported (65.2%) due to their visit to the clinic. Some (38.2%) reported using the emergency room “a great deal” less, 39.4% reported being “a great deal” more aware of what they could do to improve their situation, and 42.4% reported having a better understanding of their situation. After providing ePROs to their nurses over time, there was no significant difference in patients’ reports of change (.98 at Time 1, 1.02 at Time 2; *t* (61) = −.214, *p* = .83), nor did they perceive difference in their received care (.73 at Time 1, .92 at Time 2; *t* (62) = −1.23, *p* = .22). Specifically related to the item pertaining to satisfaction with care received, there was no significant difference on level of satisfaction over time (3.92 at Time 1, 3.88 at Time 2) following the use of ePROs (*t* (64) = 0.83, *p* = .41).

**Table 4:** Patients’ reports on the impact of using ePRO data

Question	A great deal	Moderately	Minimally	Just the same	Not as good
As a result of working with your nurse from the home dialysis clinic, has anything changed for you?	31.8%	19.7%	16.7%	31.8%	0%
As a result of having your quality of life scores available to your nurse from the home dialysis clinic, has anything changed for you?	7.7%	30.8%	20%	41.5%	0%
Was your experience today, having your quality of life scores available to your nurse from the home dialysis clinic, any different from the care you received in the past?	9.2%	15.4%	30.8%	44%	0%
As a result of my visit to the home dialysis clinic:					
I am healthier	31.8%	27.3%	10.6%	30.3%	0%
I feel more hopeful	31.8%	33.3%	7.6%	27.3%	0%
I feel more supported	65.2%	13.6%	3%	18.2%	0%
I have a better understanding of my situation	42.4%	37.9%	6.1%	13.6%	0%
I am more aware of what I can do to improve my situation	39.4%	37.9%	12.1%	10.6%	0%
I use the emergency room less	38.2%	7.3%	7.3%	47.3%	0%

## Discussion

In summary, the five themes that emerged from the interviews with the nurses include: enhancing focus of the nurses, directing interdisciplinary follow-up, offering support to patients through the process, interpreting results from the visual display, and integrating into workflow. Scores on the Client Questionnaire suggested that patients believed that they received excellent care (97%), and that the nurses perfectly understood their needs (90.9%). Although there was no statistically significant change in patient satisfaction scores over time, 38.5% of the patients reported moderate or a great deal of change as a result of the use of ePROs.

Unfortunately, we did not explore the nature of those changes. There are many contextual, demographic, and clinical factors that may have influenced satisfaction. Other researchers have pointed out that satisfaction scores usually tend to be positively skewed because respondents fear that the results will reflect badly on their care providers [13]. Given the longstanding relationships between nurses and patients in these outpatient settings, patients may indeed have felt protective of their nurses. Effective interprofessional care may have also influenced these perceptions [40, 41].

Despite the assumption that provision of PROs in routine clinical practice may lead to improved patient satisfaction [42], there is little evidence that satisfaction increases when PROs are fed back to healthcare providers [16, 43, 44]. This study suggests that use of ePROse may have an impact for some patients. This should be explored further in future studies.

It has previously been found that people with kidney disease find it difficult to discuss some topics [45, 46], and some of the items in the ePROs, such as feelings of well-being, health, depression, being a burden to one’s family, dependence on healthcare practitioners, and stress caused by kidney disease may be difficult to discuss [45, 46]. Wolpert et al. [13] noted that patients were unwilling to reveal their ratings of some areas via PROs. In this study, nurse participants believed that providing a numerical score on an ePRO was perceived as safer by patients and facilitated deeper discussion. It would be important to explore this perception with patients to see if they also perceive that the ePROs enhance discussion.

Wolpert et al. [13] also reported that patients thought that their PRO instruments (PedsQL and a symptom checklist) did not fully capture patient concerns, but perhaps their measures were less comprehensive than those used in our study. While patients in this project were not asked about willingness to reveal their ratings, most (90.9%) reported that nurses perfectly understood the kind of help that they wanted. Other researchers [4, 5] support the reports of nurses in this study who believed that ePROs increased patients’ willingness to report sensitive information.

Our findings suggest that ePROs can be valuable tools to facilitate engagement of patients in discussions about their health and well-being. Further, the process of completing the questionnaires facilitated dialogue that was perceived by nurses to provide support to patient participants. It may be that patients’ experiences of satisfaction were influenced by these experienced nurses who appropriately focused on the patients rather than the technology. The nurses’ prioritization of patients’ needs and use of the reports in the interdisciplinary team follow-up may have also influenced patient’s reports of health, and fostered better understanding and awareness of the patients’ needs. Previous research has identified that renal patients desire engagement in their own care through a person-centered approach [23, 25].

Future researchers and practitioners planning to provide ePROs in real-time may want to consider the visual display of data. Nurses in this study emphasized that the visual (versus numerical) display enhanced efficiency and use of their time. While other researchers have identified that integration of ePROs requires upfront planning and training [3, 8, 46], use of ePROs has been found to be more economical in terms of time and resources [5, 6, 8, 46–50]. Integration of ePROs into the clinicians’ workflow is essential, not only for buy-in and support by practitioners, but also to avoid duplication of data entry. Further education on use of ePROs in practice, perhaps drawing on clinical guidelines where they are available, may be useful to obtain more positive outcomes. Designing longitudinal displays of ePRO data may also facilitate assessment of change over time.

This study has a number of limitations. We had a relatively small sample size and did not collect baseline patient satisfaction data. The descriptive design does not enable one to draw causal inferences. Patient participants were also not interviewed about the benefits of use of ePROs. Such interviews could yield rich data about the way that ePROs could change patient care. The patient responses were collected in an open waiting room, so the presence or comments of others (family members or other patients) may have influenced the responses.

Different questions were asked of patients and nurses regarding perceived usefulness of ePROs, so that may explain why there were different perceptions regarding utility of the measures. The nurses also had limited time (meeting with a patient once every three months) for nursing interventions to impact patient care. Limited training was provided to nurse participants on use of ePROs in clinical practice and it may be that they, or members of the interprofessional team, did not fully understand the ePRO results. Further training of the entire team on how to respond to scores may result in different outcomes.

## Conclusion

Nurses reported that sharing ePRO data in real-time informed their practice. Although patients did not report any change in satisfaction with care over time, some reported that use of ePROs changed their care. As use of PROs is growing, further work is needed to provide guidance about how and why use of this type of data can enhance person-centered care. Replication of the study in other sites would help build our knowledge base about use of ePROs in clinical practice. Further study of the process of integration of ePROs into nurses’ or other professionals’ practice over time would help guide the planning of similar processes. A randomized controlled trial allocating patients into different groups may be helpful in assessing whether use of ePROs provides documented benefits over other traditional means of patient assessment in offering quality care and improving patient outcomes.

## Abbreviations

CKD: Chronic kidney disease
ePRO: Electronic patient reported outcome
ESAS-r:Renal: Revised Edmonton Symptom Assessment System for renal patients
HRQOL: Health-related quality of life
KDQOL-36: Kidney Disease Quality of Life-36
PRO: Patient reported outcome
QOL: Quality of life
